# Promoting early neovascularization by allotransplanted adipose-derived Muse cells in an ovine model of acute myocardial infarction

**DOI:** 10.1371/journal.pone.0277442

**Published:** 2023-01-20

**Authors:** Martha G. Castillo, Tomás M. Peralta, Paola Locatelli, Candela Velazquez, Yamila Herrero, Alberto J. Crottogini, Fernanda D. Olea, Luis A. Cuniberti

**Affiliations:** 1 Instituto de Medicina Traslacional, Trasplante y Bioingeniería (IMETTYB)—Universidad Favaloro—CONICET, Ciudad de Buenos Aires, Buenos Aires, Argentina; 2 Instituto de Biología y Medicina Experimental—CONICET, Ciudad de Buenos Aires, Buenos Aires, Argentina; Indiana University School of Medicine, UNITED STATES

## Abstract

**Background:**

Recent preclinical studies have demonstrated that bone marrow (BM)-derived Muse cells have a homing mechanism to reach damaged cardiac tissue while also being able to reduce myocardial infarct size and improve cardiac function; however, the potential of BM-Muse cells to foster new blood-vessel formation has not been fully assessed. Up to date, adipose tissue (AT)-derived Muse cells remain to be studied in acute myocardial infarction (AMI). The aim of the present study was to analyze *in vitro* and *in vivo* the neovascularization capacity of AT-Muse cells while exploring their biodistribution and differentiation potential in a translational ovine model of AMI.

**Methods and results:**

AT-Muse cells were successfully isolated from ovine adipose tissue. In adult sheep, one or more diagonal branches of the left anterior descending coronary artery were permanently ligated for thirty minutes. Sheep were randomized in two groups and treated with intramyocardial injections: Vehicle (PBS, n = 4) and AT-Muse (2x10^7^ AT-Muse cells labeled with PKH26 Red Fluorescent Dye, n = 4). Molecular characterization showed higher expression of angiogenic genes (*VEGF*, *PGF* and *ANG)* and increased number of tube formation in AT-Muse cells group compared to Adipose-derived mesenchymal stromal cells (ASCs) group. At 7 days post-IAM, the AT-Muse group showed significantly more arterioles and capillaries than the Vehicle group. Co-localization of PKH26+ cells with desmin, sarcomeric actin and troponin T implied the differentiation of Muse cells to a cardiac fate; moreover, PKH26+ cells also co-localized with a lectin marker, suggesting a possible differentiation to a vascular lineage.

**Conclusion:**

Intramyocardially administered AT-Muse cells displayed a significant neovascularization activity and survival capacity in an ovine model of AMI.

## Introduction

Ischemic heart disease is a common event that greatly compromises the overall health as a result of impaired cardiac function [1]. Its most severe complication, myocardial infarction, results in a fibrotic scar which, according to its extent, leads to left ventricular remodeling and heart failure. Over the past decades, there has been a strong interest in developing treatments to regenerate the infarcted heart [2]. Stem cell therapies are regarded as a promising opportunity because of their potential ability to promote tissue regeneration by cellular differentiation, angiogenesis, matrix remodeling and immunomodulation, among others [3, 4]. In animal models, cell therapies employing mesenchymal stem cells (MSCs) and cardiac-derived cells (CDCs) have shown significant infarct size limitation and cardiac function improvement. However, in randomized clinical trials these positive effects have been subtle, and of little or no clinical relevance. One of the main reasons for these disappointing results is that the potential of these cells is greatly limited by the harsh environment of the ischemic heart, as documented by the low survival rate and engraftment of transplanted cells [5–7].

Recently, a new MSCs sub-population known as multilineage-differentiating stress-enduring (Muse) cells has been described [8]. Muse cells are highly stress-tolerant and express pluripotency markers like *NANOG*, *OCT4*, and *SOX2*; moreover, they are able to differentiate into endodermal, mesodermal, and ectodermal lineages [8]. These cells are located in most tissues in a quiescent state and are activated by cellular stress [9]. Muse cells can be isolated from dermal fibroblasts, bone marrow (BM-Muse), adipose tissue (AT-Muse) among other tissues by fluorescence-activated cell sorting or under extreme cellular stress conditions [10]. BM-Muse cells have a higher expression of ectodermal and endodermal markers whereas Muse cells derived from adipose tissues have higher expression of mesodermal markers [11]. Noteworthy, Muse cells have low immunogenicity and the ability to promote neovascularization by mechanisms involving cell differentiation [12–14]. The migration of BM-Muse cells to damaged tissue involves a homing mechanism mediated by sphingosine monophosphate (S1P) [12]. In addition, a recent clinical study has shown that patients with an increased number of circulating Muse cells after AMI had an improvement in ventricular function and less remodeling [15]. Up to date, the neovascularization potential of adipose-derived Muse cells has not been studied in AMI. The aim of the present study was to analyze *in vitro* and *in vivo* the neovascularization capacity of AT-Muse cells while exploring their biodistribution and differentiation in a translational ovine model of AMI.

## Materials and methods

### Isolation of ovine Muse cells

Adipose tissue was collected sterilely from donor Corriedale male sheep weighing ~30 kg as described in the animal model section. Muse cells were isolated under a stress procedure as described previously with minor modifications [16, 17]. Adipose Tissue was cut into small pieces, rinsed twice with PBS and then incubated with (0.1% (wt/vol) collagenase (Merck, Kenilworth, NJ, USA, cat. #17100017) in DMEM low glucose (Gibco, Waltham, MA, USA, cat. #31600034) for 60 minutes at 37°C with constant agitation. Successively samples were incubated overnight at 4°C and 50 rpm. The next day, the samples were centrifuged at 1500 rpm and the supernatant was discarded, and the pellet was incubated with red blood lysis buffer and washed twice with PBS. Isolated cells were cultured in low glucose DMEM (1g/L) (Gibco, Waltham, MA, USA), 20% SFB (Natocor, Cordoba, Argentina), 1% ATB (Gibco, Waltham, MA, USA) and incubated at 37% with 5% CO_2_ in p60 non-adherent plates (Greiner Bio-One, Frickenhausen, Germany), each one with 3,5x10^6^ cells. The culture medium was changed after 48 hours; in addition, 5 days after isolation the cells were cultured in adherence bottles and sub-cultured until passage 4 and 80% confluence for the subsequent experiments. Adipose-derived mesenchymal stromal cells (ASCs) were also extracted from the same donors as control as we reported previously [18].

### Flow cytometry characterization of ovine Muse cells

AT-Muse cells were incubated with the following monoclonal antibodies: Rat Anti-Human SSEA3 (Invitrogen, Waltham, MA, USA, cat. # MA1-020-D488), Mouse Anti-sheep CD44 (cat. # MCA2219F), Mouse Anti-sheep CD45 (cat. # CD45 MCA2220F), (both from AbD Serotec, Raleigh, NC, USA) and mouse-anti-humanCD166 (BD Biosciences, Franklin Lakes, NJ, USA, cat. #559263) and characterized by flow cytometry (FACS Calibur, BD Biosciences, Franklin Lakes, NJ, USA). Data was acquired and processed with Cyflogic 1.2.1 software (CyFlo Ltd, Turku, Finland).

### Gene and protein expression analysis

RNA from AT-Muse cells, ASCs at passage 4 (P4) and heart tissue was extracted with Trizol reagent (Invitrogen, Waltham, MA, USA, cat. #15596026) according to the manufacturer’s instructions. Quantitative real-time PCR (RT-qPCR) (Step One, Applied Biosystems, Waltham, MA, USA) was performed using Sybr Green (Applied Biosystems, cat. #4472908). The oligonucleotides used were NANOG Homeobox (*NANOG*) (Frw5´-CCCCGAAGCATCCAACTCT-3, Rev5´-TGCAAGGACGCGTAACTTTCT–3´), POU Class 5 Homeobox1 (*OCT4*) (Frw5´-TCTGCCGTTTTGAGGCTTTG-3´, Rev5´-GTCTCTGCCTTGCATATCTCCT-3´), SRY-Box Transcription Factor 2 (*SOX2*) (Frw5´-TCAGATGCAGCCCATGCA-3´, Rev5´-GTCTGCGAGCTGGTCATAGAGTT-3´), Sphingosine-1-Phosphate Receptor 2, (*S1PR2*) (Frw5´-TGCTCTACCAGGCCCACTACTT-3´, Rev5´-CGGGTTCAACAGCGAGTTG-3´), Vascular Endothelial Growth Factor (*VEGF*), (Frw5´-GCCCACTGAGGAGTTCAACATC-3´, Rev5´-GCTGGCTTTGGTGAGGTTTG-3´), Angiogenin (*ANG*) (Frw5´-TGCCCATTTCTGCAGACTTGT-3´, Rev5´-GGCTCAAGACCATGACCATGT-3´) and Placental Growth Factor (*PGF*) (Frw5´-CCCTGGAGACAGCCAACGT-3´, Rev 5´-GGCTGGTCCAGAGAGTGGTACT-3´). Relative gene expression was performed using the 2-ΔΔct method and Glyceraldehyde-3-Phosphate Dehydrogenase (*GAPDH*) was used as an endogenous control for normalization (Frw5´-TCTTCCAGGAGCGACATCC-3´, Rev 5´-TGAGCCCCAGCCTTCTCC-3´).

For protein analysis, samples from cell culture (AT-Muse and ASCs) and heart tissue were treated with lysis buffer (20 mM Tris–HCl pH 8, 137 mM NaCl, 1% Nonidet P-40 and 10% glycerol) supplemented with protease inhibitors (0.5 mM PMSF, 0.025 mMN-CBZ-l-phenylalanine chloromethyl ketone, 0.025 mMN-p-tosyl-lysine chloromethyl ketone and 0.025 mM l-1-tosylamide-2-phenyl–ethylchloromethyl ketone). The lysates were centrifuged at 10000g for 10 min at 4°C. Protein concentration was measured by the Bradford assay. After boiling for 5 min, 20 μg of protein was applied to a 10% SDS–polyacrylamide gel, and electrophoresis was performed at 25 mA for 1.5 h. The resolved proteins were transferred for 2 hs. onto nitrocellulose membranes. The blot was preincubated in blocking buffer (5% nonfat milk, 0.05% Tween 20 in 20 mM TBS pH 8.0) for 1 h at room temperature and incubated overnight in blocking buffer at 4°C with diluted primary antibodies as follows: rabbit anti-human VEGF 1:1000 (Abcam, Cambridge, UK, cat.# ab46154); mouse anti-human CD34 1:100 (Santa cruz biotechnology, Dallas, TX, USA, cat. # SC74499) and rabbit anti-bovine GAPDH 1:8000 (Cell Signaling Technology, Inc., Danvers, MA, USA, cat.# 2118). The immunoblots were then incubated with HRP-conjugated secondary antibodies, namely goat anti-rabbit 1:1000 (Sigma Aldrich, Burlington, MA, EE. UU, cat. # A4914) or goat anti-mouse 1:1000 (R&D Systems, MN, USA, cat# HAF007), as required. Signal was detected by chemiluminescence. Protein levels were analyzed by densitometry and each band was normalized to the density of GAPDH as an internal control. Densitometry analysis was realized with Scion Image for Windows (Scion Corporation, Worman’s Mill, CT, USA). Optical density data are expressed as arbitrary units ± SD. All blots shown were representative of at least three independent experiments.

### Tube formation assay

Conditioned medium was harvested from AT-Muse cells and ASCs at P4 by centrifugation at 1500 rpm x 5 min and stored at -80°C until use. Human Mammary Epithelial (HMEC) cells were seeded on a matrigel layer (Gibco, Waltham, MA, USA, cat. # A1413202), in 96-well plates and conditioned medium from each of the samples was added and incubated for 6 hours at 37°C and 5% CO_2_. At the end of the experiment, the supernatant of each well was discarded and each sample was fixed with 4% paraformaldehyde. Random fields were photographed on the Cytation 5 (Agilent Technologies, Santa Clara, CA, USA) at 25x magnification to then be analyzed and counted in the Image J Software (U. S. National Institutes of Health, Bethesda, Maryland, USA). Rings were counted per unit area in mm^2^.

### Animal model and cell transplantation

All animal procedures were performed following the Committee for the Update of the Guide for the Care and Use of Laboratory Animals [19] and under approval and monitoring of the Institutional Committee for the Care and Use of Laboratory Animals (CICUAL) of the Favaloro University (NIH-OLAW Animal Welfare Assurance Number F16-00126/A5556-01) 2021–003 SIUF 2021 (Buenos Aires, Argentina).

Eight male sheep were premedicated with 0,5 mg/kg acepromazine maleate; anesthesia was induced with 3mg/kg propofol and maintained with 2% isoflurane in oxygen under mechanical ventilation. A left thoracotomy was performed at the 4th intercostal space under sterile conditions and electrocardiographic and oximetry monitoring. The pericardium was opened and one or more diagonal branches of the left anterior descending artery were ligated to generate an anterior or anterolateral LV wall infarction involving ~20–25% of the LV mass. Sheep were randomized by veterinarians, who were also the only ones aware of group allocation, into 2 groups: Vehicle (PBS, n = 4) and AT-Muse (2x10^7^ ovine Muse cells labeled with PKH26 Red Fluorescent Dye for in vivo tracking, n = 4). PKH26 was employed according to the manufacturing instructions (Sigma Aldrich, Burlington, MA, EE. UU, cat. # PKH26GL-1KT). The dose and sample size were defined according to previous experiences in our laboratory [20, 21] and in accordance with a recent systematic review [22]. Thirty minutes after permanent ligation, all animals received 10 intramyocardial injections in the infarct border zone (final volume of 2 ml). Finally, the thorax was closed by layers and the animal was extubated and returned to the animal house under analgesic and antibiotic medication. None of the animals died during or after the procedure. The nature of the injections was blinded to the investigators processing the samples and performing data analysis. At 7 days post-AMI, sheep were euthanized by an intravenous overdose of propofol (15 mg/kg) followed by a bolus injection of potassium chloride (60 mEq) to arrest the heart in diastole. Samples of remote organs (liver, spleen, retina, lung, kidney, and gonad) were extracted and stored at -80°C until use. The left ventricle was opened along the posterior interventricular sulcus, the endocardium was exposed and samples were obtained from 3 representative zones: the center of the AMI, its border and an area remote to the site of infarction. Samples were fixed in 4% paraformaldehyde for 24 hours and included in paraffin or included directly in cryoplast for histological and immunofluorescence analysis, respectability. Samples were frozen in liquid nitrogen and stored at -80°C for molecular biology studies.

### Histological analysis

For immunohistochemistry, primary monoclonal antibodies used were mouse anti- human smooth muscle actin (Biogenex, Fremont, CA, USA, cat. # AM128-5M), rabbit anti-human Ki67 (Ventana, Oro Valley, AZ, USA, cat. # 790–4286), and biotinylated lycopersicon esculentum Lectin (Vector Laboratories, Burlingame, CA, USA, cat. # B-1175). Ten to fifteen random fields were photographed in an optical microscope (Carl Zeiss, Axio Observer 7, Oberkochen, Germany).

For immunofluorescence, the following antibodies were used: mouse anti-human sarcomeric actin (Dako, Santa Clara, CA, USA, cat. # M0874), mouse anti-human desmin (cat. # NCL-L-DES-DERII), mouse anti-human troponin T (both from Leica, Wetzlar, Germany, cat. # NCL-TROPT), rabbit anti-human anti-connexin 43 (Santa cruz biotechnology, Dallas, TX, USA, cat. # 101660), biotinylated lycopersicon esculentum Lectin (Vector Laboratories, Burlingame, CA, USA, cat. # B-1175) and mouse anti-human smooth muscle actin (Biogenex, Fremont, CA, USA, cat. # AM128-5M) antibodies were used, and a secondary goat anti-mouse antibody conjugated with FITC (Kallestad, Chaska, MN, USA, cat. # 400288) and goat anti-rabbit conjugated with FITC (Vector Laboratories, Burlingame, CA, USA, cat. # FI-1000) were added. Anti-Biotinylated Lectin was labeled with FITC-conjugated streptavidin (Vector Laboratories, Burlingame, CA, USA, cat. # SA-5001).

Direct immunofluorescence was performed, incubating AT-Muse cells at P4 with primary rat anti-human SSEA3 conjugated with FITC (Invitrogen, Waltham, MA, USA, cat. # MA1-020-D488). In all cases, DAPI ready-made solution (Merck, Kenilworth, NJ, USA, cat. # MBD0015-10ml) was used to dye nuclei. Photographs were taken with an inverted fluorescence microscope (Carl Zeiss, Axio Observer 7, Oberkochen, Germany) and cells were counted individually in each cross-section with ImageJ software [23]. In all cases, the specificity controls of the secondary antibody (without primary) and auto-fluorescence (without any antibody) were performed.

### Statistical analysis

Data was analyzed employing Mann-Whitney U test, Student t-test, and two-way ANOVA with Tukey´s post-hoc test after assumption assessment. Results were expressed as mean ± standard deviation. Values of p<0.05 were considered indicative of statistically significant differences. Analyses were performed using GraphPad Prism software version 6.0 (GraphPad Software Inc.).

## Results

### 1 Isolation and characterization of AT-Muse cells

**Fig 1:**
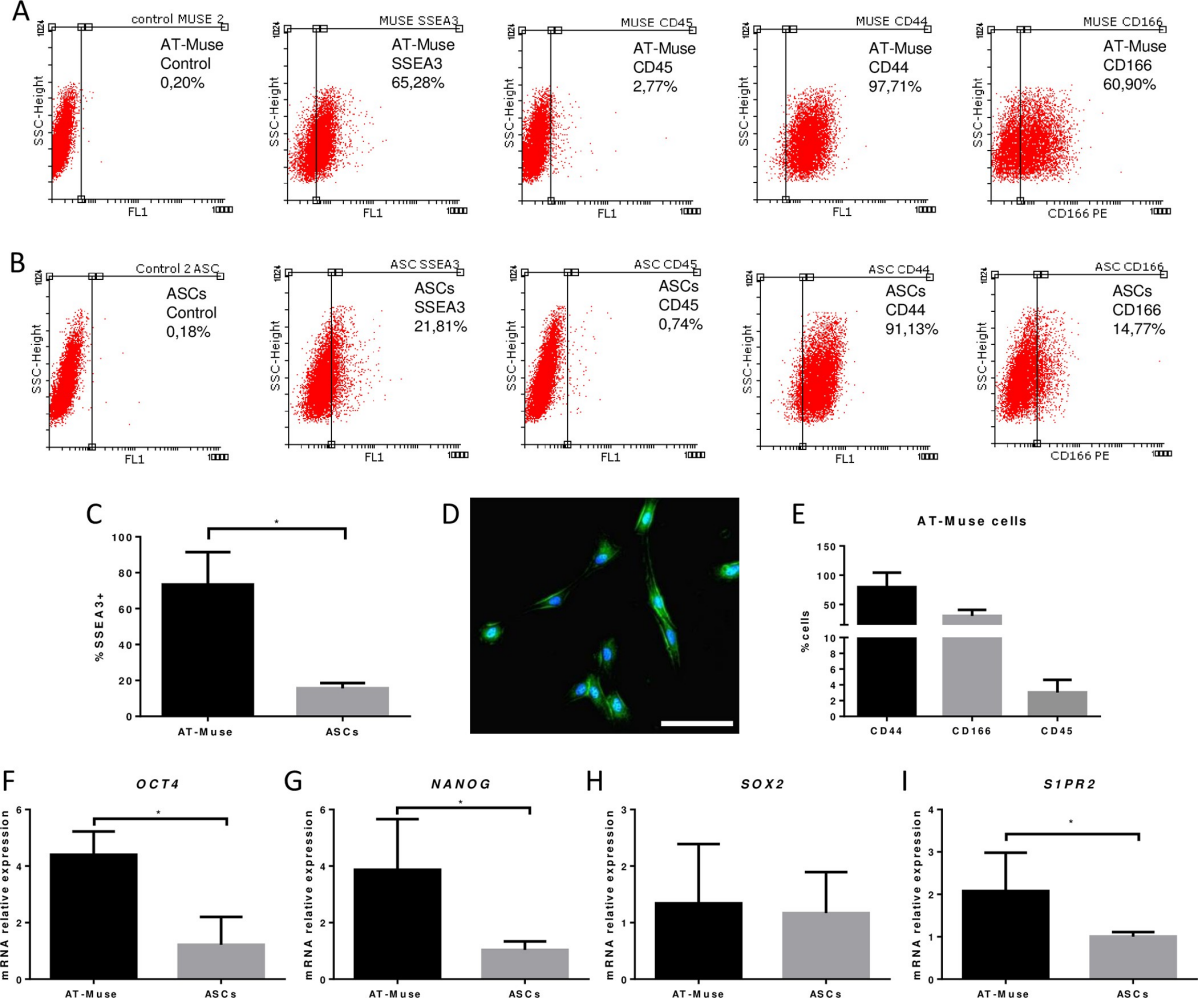
Flow cytometry and gene expression analysis. Labeling of A: AT-Muse cells and B: ASCs with antibodies anti- SSEA3, CD45, CD44, CD166 and controls. C: The % of SSEA3+ cells was higher in the AT-Muse group than in the ASCs group. D: Immunofluorescence image confirming the labeling of SSEA3 (green) and DAPI (blue) in AT-Muse cells at P4 *in vitro*. Scale bar = 100 μm. E: AT-Muse cells were CD166+, CD44+ and CD45- confirming their immunophenotype. F-I: The AT-Muse group showed higher expression of *OCT4*, *NANOG* and *S1PR2* than the ASCs group. Results are means ± SD. *p<0.05, Mann-Whitney U test.

AT-Muse cells were isolated from abdominal fat tissue (30–50 cm^3^) of donor sheep with an average yield of 1.4x10^5^ cells/cm^3^. ASCs were identified with the marker profile CD44+ (69.5%±17.3%), CD166+ (6.9%±3.7%) and CD45- (1.1%±0.3%). AT-Muse cells were CD44+ (79.7%±24.7%), CD166+ (30.1%±10.5%) and CD45- (3.0%±1.6%) while also showed an increased expression of the SSEA3+ embryonic marker when compared to ASCs (73.4%±18.0% vs. 15.6%±2.9%; p < 0,05) (Fig 1A–1C and 1E). Negative expression was considered as a ratio ≤ 3%. Morphological analysis by fluorescence microscopy showed a spindle-shaped fibroblast-like morphology for adherent AT-Muse cells at P4 (Fig 1D). Gene expression analysis showed significant expression of the pluripotency markers *OCT4* (4.4±0.8 vs. 1.2±1.0; p<0.05), *NANOG* (3.9±1.8 vs. 1.0±0.3; p<0.05), and the homing receptor *S1PR2* (2.1±0.9 vs. 1.0± 0.1; p<0.05) in the AT-Muse cells group compared to the ASCs group at P4 (Fig 1F–1I).

### 2 Angiogenic gene expression and tube formation assay of AT-Muse cells

**Fig 2:**
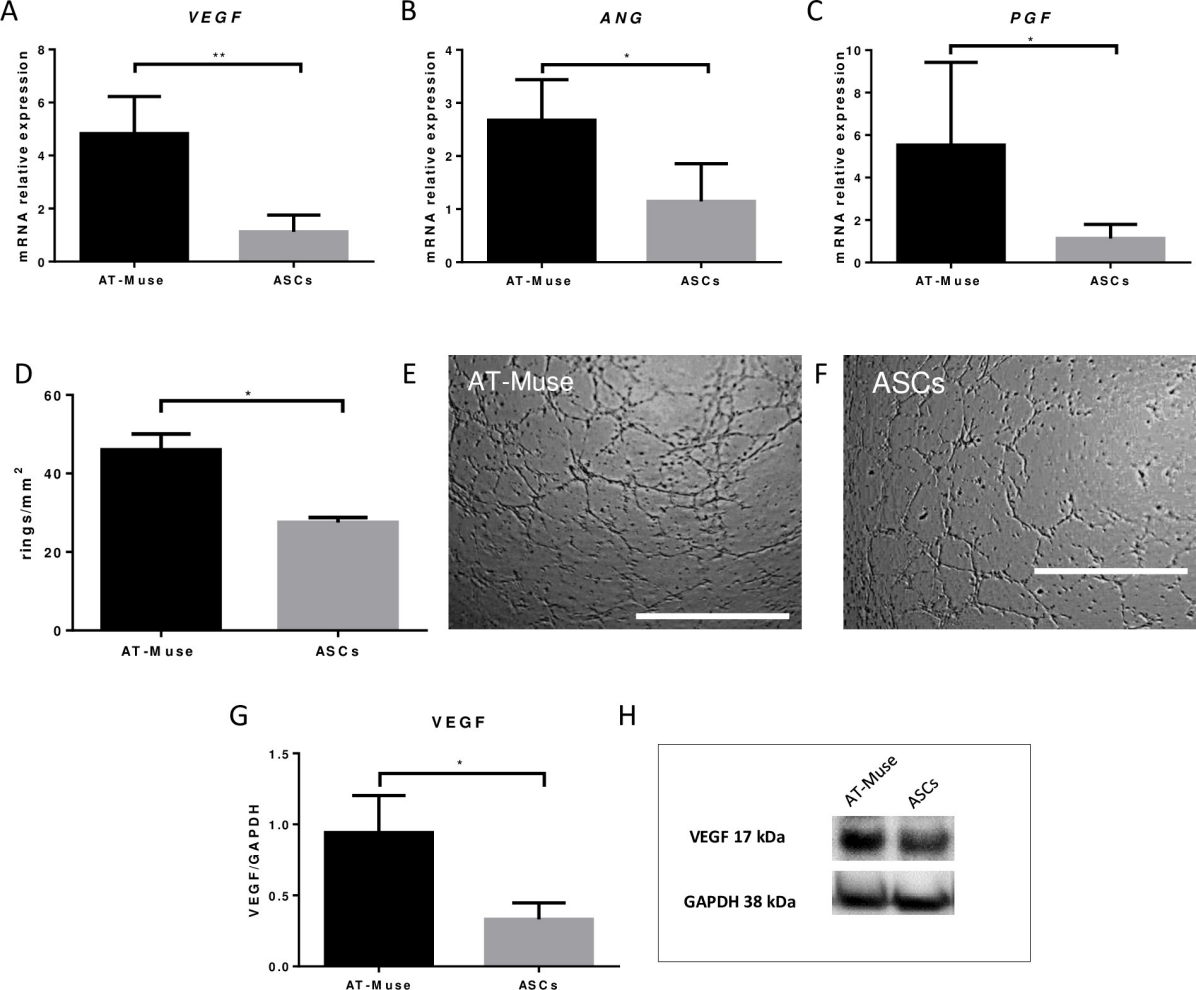
Angiogenic potential of AT-Muse cells. A-C: *VEGF*, *ANG*, and *PGF* gene expression was higher in the AT-Muse group than in the ASCs group. D: Tube formation assay: the AT-Muse group showed an increased number of rings per area (mm^2^) compared to the ASCs group. E-F: Representative images of rings formed in the AT-Muse and ASCs groups. G-H: VEGF protein expression was higher in the AT-Muse group than in the ASCs group. Scale bar 1000 μm. Results are mean ± SD. ** p < 0.01; *p < 0.05, Mann-Whitney U test.

The angiogenic genes *VEGF* (4.8±1.4 vs. 1.1±0.6; p<0.01), *ANG* (2.7±0.8 vs. 1.1±0.7; p<0.05) and *PGF* (5.5±3.9 vs. 1.1±0.7; p<0.05) showed a significantly higher expression in the AT-Muse group compared to the ASC group at P4 (Fig 2A–2C). The tube formation assay revealed significant differences in the number of rings per area in the AT-Muse group compared to the ASC group (45.9± 4.1 vs. 27.6±1.4 rings/mm^2^; p<0.05) (Fig 2D–2F). Consistently, VEGF protein expression was significantly higher in the AT-Muse group compared to the ASCs group (0.9±0.3 vs. 0.3±0.1; p<0.05) (Fig 2G and 2H).

### 3 In vivo studies at 7 days post AMI

#### 3.1 Engraftment and differentiation of transplanted AT-Muse cells

**Fig 3:**
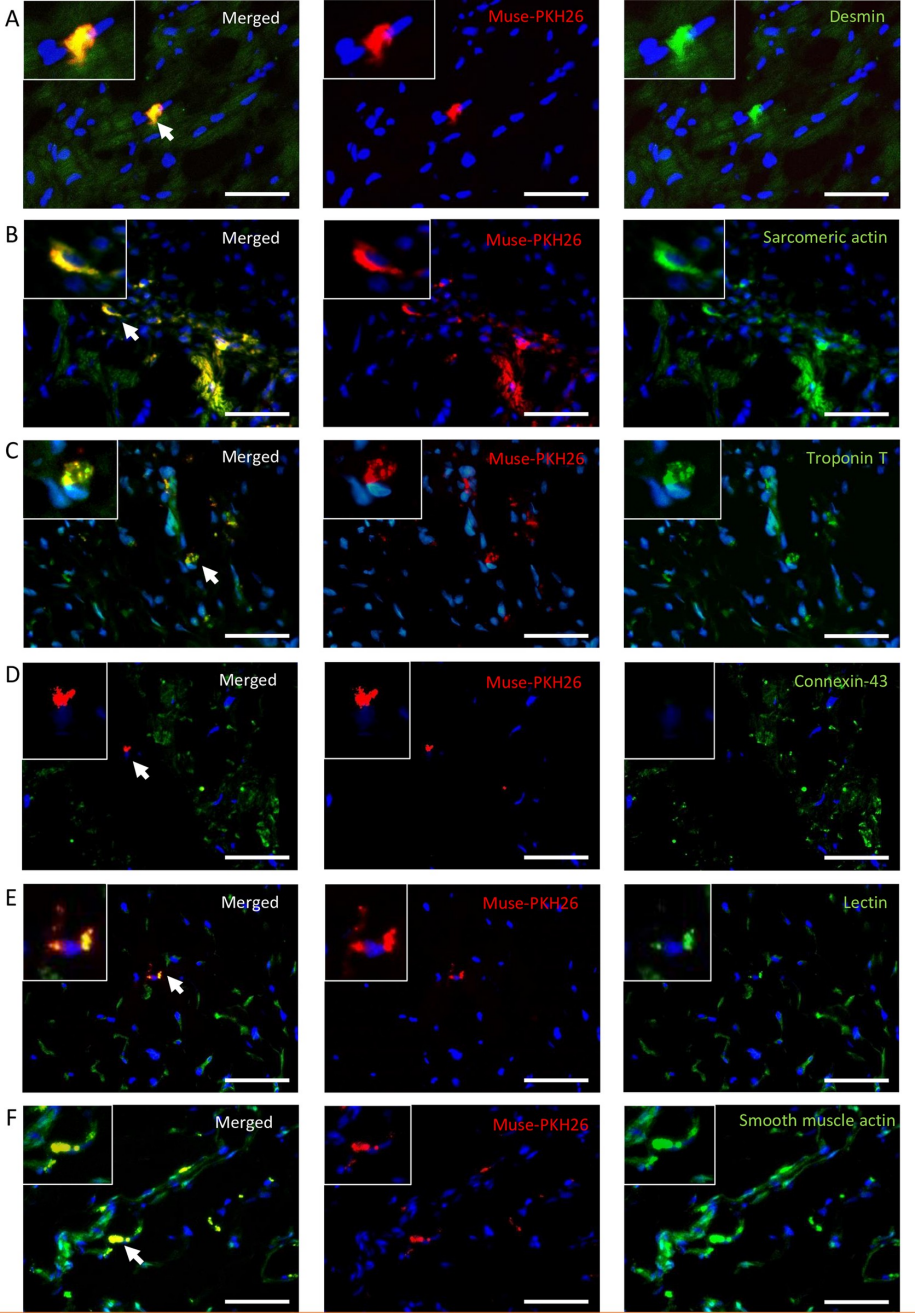
Differentiation of PKH26+ cells. A-F: Fluorescence microscopy analysis revealed the presence of PKH26+ cells at 7 days post-AMI in the infarct border zone of AT-Muse group. A: PKH26+ Muse cells (red), Desmin (green), and DAPI (blue) are merged. B: PKH26+ Muse cells (red), Sarcomeric actin (green), and DAPI (blue) are merged. C: PKH26+ Muse cells (red), Troponin T (green), and DAPI (blue) are merged. D: PKH26+ Muse cells (red), Connexin-43 (green), and DAPI (blue) are merged. E: PKH26+ Muse cells (red), Lectin (green), and DAPI (blue) are merged. F: PKH26+ Muse cells (red), Smooth muscle, actin (green) and DAPI (blue) are merged. One cell is enlarged in the top left box (white arrow). Bars = 50 μm.

PKH26+ cells were mainly found in the infarct border zone of the AT-Muse group (350.0±96.3 vs. 0.3±0.2 PKH26+ cells/cross-section; p <0.001). The co-localization of PKH26+ cells with the markers desmin (20.0%±6.9%), sarcomeric actin (22.3%±8.6%), and troponin T (14.3%±5.6%) implied the differentiation efficiency of some Muse cells to a cardiac fate (Fig 3A–3C), while no co-localization was observed with the connexin-43 marker (Fig 3D); moreover, PKH26+ cells also co-localized with the marker lectin (13.2%±2.9%), suggesting a possible differentiation to a vascular lineage (Fig 3E) mainly forming part of newly formed blood vessels due to their thin marking of smooth muscle actin (Fig 3F).

#### 3.2 Neovascularization and cellular proliferation

**Fig 4:**
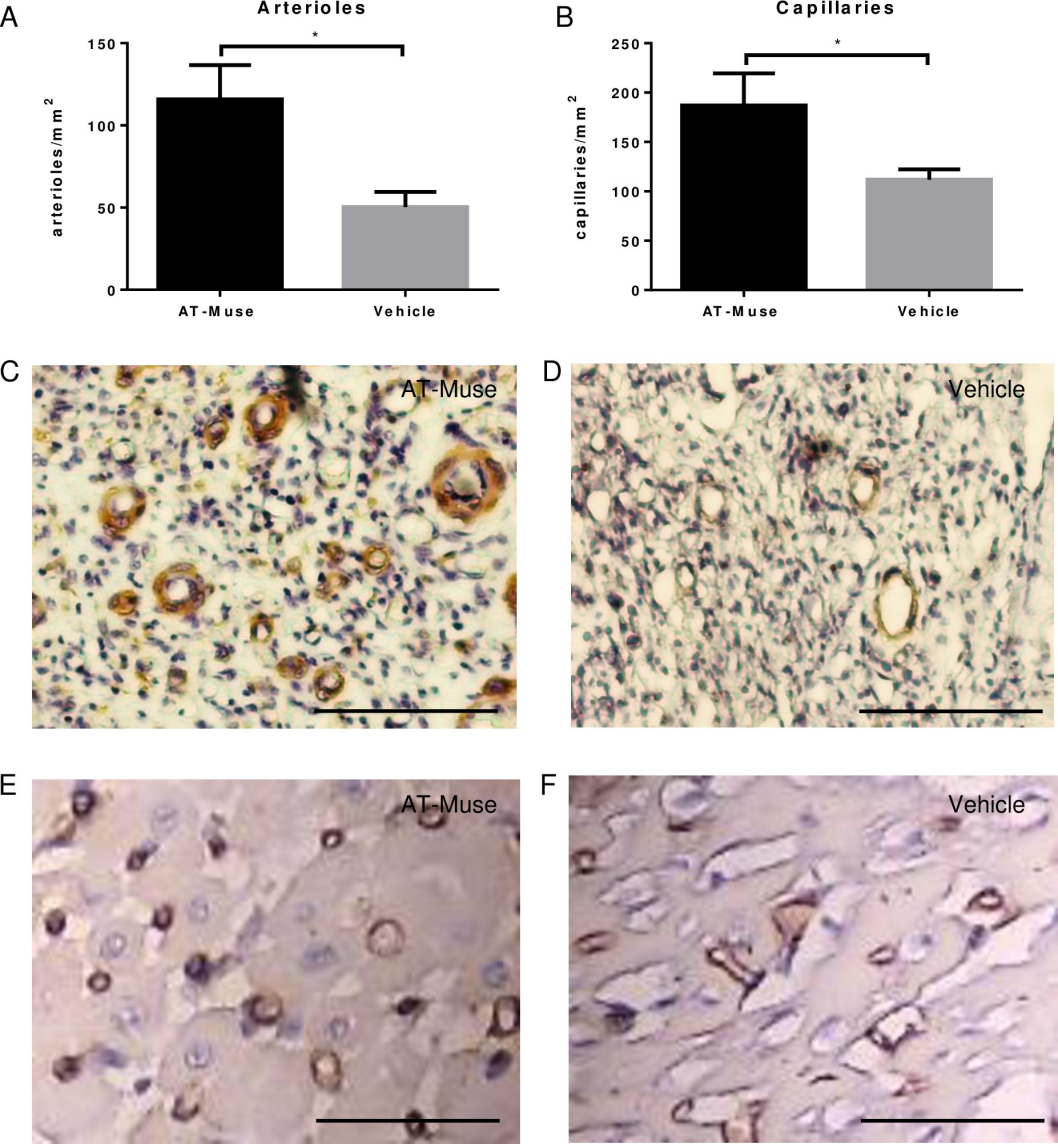
Microvascular density at 7 days post-AMI. A-B: Arteriolar and capillary densities were significantly higher in the AT-Muse group than in the Vehicle group. C-D: Representative slices of the infarct border zone for the AT-Muse and Vehicle groups. Arterioles were stained with an anti-smooth muscle actin antibody and counterstained with hematoxylin (bars = 100 μm). E-F: Representative slices of the infarct border zone for the AT-Muse and Vehicle groups showing capillaries stained with a biotinylated lectin and counterstained with hematoxylin (bars = 100 μm). Results are means ± SD. *p<0.05, Mann-Whitney U test.

**Fig 5:**
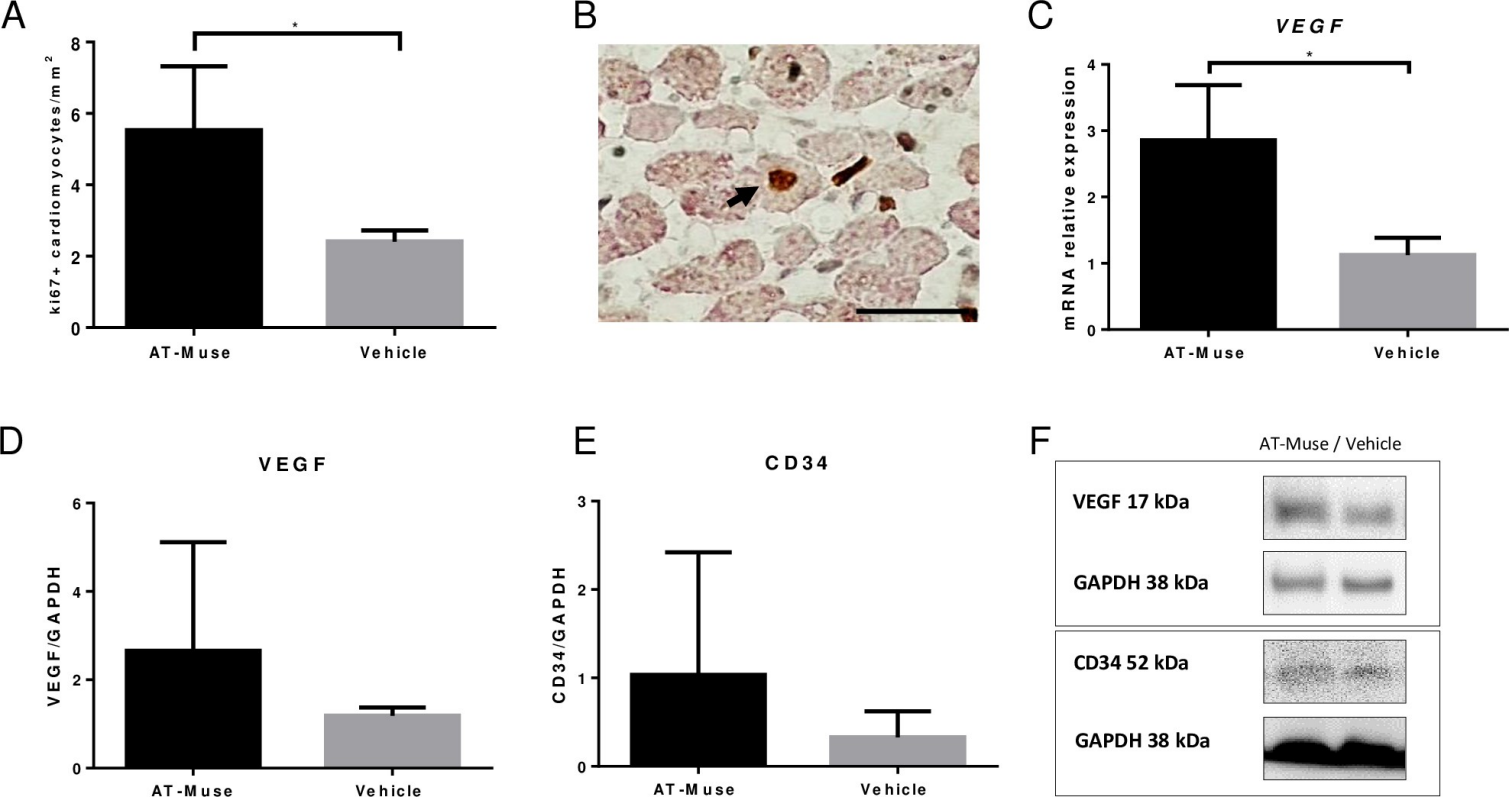
Cycling cells and protein expression of angiogenic markers at 7 days post-AMI. A: The number of Ki67+ cells in the AT-Muse group was significantly higher than in the Vehicle group. B: Representative image of Ki67+ nuclei (brown) counterstained with sarcomeric actin (violet) in the AT-Muse group (bar = 20 μm). C-D: The VEGF had higher gene and protein expression in the infarct border zones of the hearts of AT-Muse group in comparison with the Vehicle group, respectively. E: The CD34 protein expression was higher than in the Vehicle group. F: representative western blot images for VEGF and CD34. Results are means ± SD. *p<0.05, Mann-Whitney U test.

The AT-Muse group had significantly more arterioles (115.8±21.0 arterioles/mm^2^) and capillaries (187.0±32.5 capillaries/mm^2^) than the Vehicle group (50.3±9.2 arterioles/mm^2^ and 111.7±10.4 capillaries/mm^2^ respectively; p<0.05) in the infarct border zone (Fig 4A–4F). Moreover, the number of Ki67+ cells in the infarct border zone was significantly higher in the Muse group than in the Vehicle group (5.5±1.8 vs. 2.4±0.3 Ki67+ cells/mm^2^; p<0.05) (Fig 5A). Several Ki67+ cells co-localized with the sarcomeric actin marker (Fig 5B). Gene expression analysis revealed a higher expression of *VEGF* in the AT-Muse group compared to the Vehicle group (2.9±0.8 vs. 1.1±0.3; p<0.05) (Fig 5C). No significant differences were found for *ANG* (0.5±0.2 vs. 0.7±0.6; p = NS) and *PGF* (0.7±0.3 vs. 0.7±0.5; p = NS). Protein expression analysis revealed a marginal, though not significant, increase in the protein expression of VEGF (2.7±2.5 vs. 1.2±0.2; p = NS) and CD34 (1.3±1.9 vs. 0.2±0.1; p = NS) at 7 days post-AMI in the hearts that were transplanted with Muse cells compared with those in the vehicle group that did not receive treatment (Fig 5D–5F).

**Fig 6:**
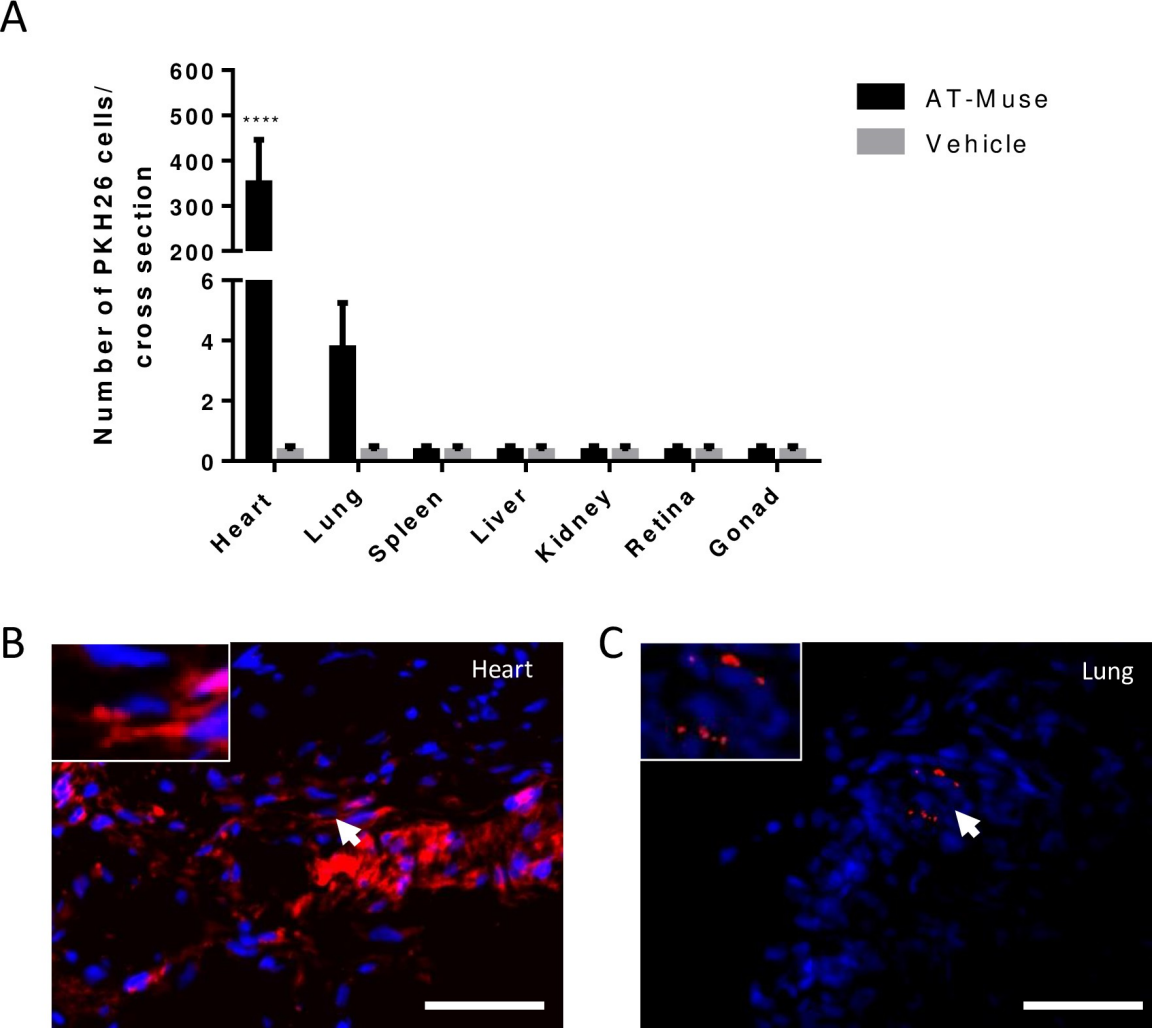
Biodistribution analysis of AT-Muse cells. A: High number of PKH26-labeled ovine AT-Muse cells were found in the infarct border cross sections. Some Muse cells were found in the lungs although no Muse cells were found in spleen, liver, kidney, retina, gonad or in the Vehicle group. Results are means ± SD; ****p<0.0001, two-way ANOVA with Tukey’s post-hoc test for multiple comparisons. B: Representative microscopic merged image of infarct border zone of heart injected with Muse, PKH26+ cells (red) and DAPI (blue). C: Representative microscopic merged image from lung tissue of AT-Muse group, PKH26+ cells (red) and DAPI (blue).

**3.3 Biodistribution of transplanted AT-Muse cells.** Biodistribution analysis showed that the number of PKH26+ cells was significantly higher in the heart of AT-Muse cells-treated animals in comparison to any other organs of the AT-Muse group and any organs -including the heart- of Vehicle-treated animals. The number of PKH26+ cells was of 350.0±96.3 per cross-section for AT-Muse hearts vs. all comparisons; p<0.0001. In addition, it was possible to observe a lesser extent of PKH26+ cells in lungs without statistical significance in the multiple comparison test (3.8±1.5 cells per cross section, p = ns) (Fig 6).

## Discussion

The regenerative potential of Muse cells has been widely assessed in cardiovascular, respiratory and gastrointestinal diseases [10]. Up to date, Muse cells have shown their regenerative features in pre-clinical and clinical studies, most of which employed bone marrow-derived Muse cells [10, 24]. The isolation and culture of BM-Muse cells is an invasive and demanding procedure; thus, a simpler method would be preferable [24]. In this study, we successfully isolated ovine Muse cells from adipose tissue by severe cellular stress obtaining a high yield of SSEA3+ cells, thus validating the stress protocol as a feasible method for processing fat samples. Moreover, our inclination towards AT-Muse cells lies in their higher expression of mesodermal markers compared to BM-Muse cells [11], a feature that makes AT-Muse cells suitable for strategies seeking to regenerate vessels and cardiomyocytes.

Our *in vitro* experiments confirmed the stemness of AT-Muse cells, as indicated by the high expression of *NANOG* and *OCT4*, and their previously reported homing capability [12, 24], on account that they expressed *S1PR2*. In addition, the *in vitro* experiments revealed the neovascularization potential of Muse cells as evidenced by the increased number of rings per area found in the tube formation assay, a result attributable to the paracrine effect of the observed gene and protein expression of *VEGF* and gene expression of *ANG* and *PGF*. These results also show that AT-Muse cells hold an advantage over ASCs for strategies aiming to regenerate lost vasculature.

For the *in vivo* experiments, we used a highly translational, large mammalian model of AMI, namely adult sheep undergoing acute, permanent coronary artery ligation. The procedure consisted of injecting at the infarct border AT-Muse cells labeled with the PKH26 red fluorescent dye, which displays low cellular toxicity and long-lasting activity up to 30 days [25, 26]. PKH26+ cells were found in the injection area, though in low numbers, a fact at least partially attributable to the injectate reflux due to cardiac contraction. Nevertheless, the differentiation potential of Muse cells was evidenced by the presence of PKH26+ cells that were simultaneously positive for troponin T, sarcomeric actin or desmin. These findings imply that some Muse cells differentiated towards a cardiac lineage, thus exhibiting *in vivo* their pluripotent nature. Interestingly, the ratios of differentiation to cardiac lineage are in range with those published by Yamada *et al*. in a rabbit model of AMI [12]. On the other hand, co-localization of PKH26 with connexin-43 at 7 days post implant was negative, suggesting lack of gap junctions between AT-Muse cells and resident cardiomyocytes. This makes unlikely that cardiomyocyte-like cells can contract and relax synchronously with the rest of the heart. Presence of connexin-43 in cardiomyocyte-like cells derived from intravenously administered Muse cells was observed by Yamada *et al*. at 14 days post treatment, suggesting that expression of gap junction genes in these cells occurs later than at one week, namely the time point at which we looked for connexin-43 expression.

The differentiation of AT-Muse cells towards a cardiac lineage could be explained by the increased *VEGF* expression in the infarct border zone of the AT-Muse group. Previous studies have demonstrated that VEGF promotes the differentiation of adipose derived stem cells (namely the ones used in our study), embryonic stem cells and human induced pluripotent stem cells into cardiomyocytes by the activation of the VEGF-Flk-1-ERK pathway [27–29].

On the other hand, the co-localization of Ki67 and sarcomeric actin indicated that some cardiomyocytes re-entered the cardiac cycle, a phenomenon probably related to paracrine factors released by nearby Muse cells. Furthermore, we found a higher expression of *VEGF* as well as an increased number of arterioles and capillaries in the ischemic border zone of the Muse group that matched our *in vitro* findings regarding neovascularization. The co-localization of PKH26+ cells with a lectin marker evidenced that the neovascularization potential of Muse cells was also mediated by their capacity to differentiate into a vascular lineage. In addition, PKH26+ cells were usually seen grafted onto blood vessels with a very thin media layer, suggesting that they were newly formed (Fig 3F).

The biodistribution of PKH26+ Muse cells was mostly focused in the heart and to a lesser extent in the lungs at 7 days post treatment, in agreement with previous results showing that cells administered intramyocardially redistribute into the lungs after primarily distribute in the myocardium [30]. Although the formation of new microvessels and the presence of cycling cardiomyocytes should be primarily attributed to the engraftment of Muse cells in the heart, a paracrine signaling of Muse cells trapped in the lungs cannot be discarded. In fact, this effect has been previously reported for the systemic administration of MSCs [31].

In summary, our results show that AT-Muse cells display significant neovascularization activity, as indicated by enhanced tube formation assay *in vitro* and increased capillary and arteriolar proliferation *in vivo*, a phenomenon likely due to a paracrine effect. Our study also illustrates the ability of AT-Muse cells to differentiate into a cardiac and vascular lineage and to stimulate the cardiomyocytes to reenter the cell cycle. Finally, the survival of AT-Muse cells in the ischemic border zone showed their capacity to endure a harsh environment. Overall, these results encourage performing further studies in larger populations at longer follow up periods to assess the effects of AT-Muse cells on cardiac function and their potential utility in ischemic heart disease.

## Supporting information

S1 File Full ARRIVE 2.0 guidelines checklist. (PDF)

S2 File Minimal underlying data set. (XLSX)
